# Yeast nitrogen utilization in the phyllosphere during plant lifespan under regulation of autophagy

**DOI:** 10.1038/srep09719

**Published:** 2015-04-21

**Authors:** Kosuke Shiraishi, Masahide Oku, Kosuke Kawaguchi, Daichi Uchida, Hiroya Yurimoto, Yasuyoshi Sakai

**Affiliations:** 1 Division of Applied Life Sciences, Graduate School of Agriculture, Kyoto University, Kitashirakawa-Oiwake, Sakyo-ku, Kyoto 606-8502, Japan; 2 Research Unit for Physiological Chemistry, The Center for the Promotion of Interdisciplinary Education and Research, Kyoto University, Kyoto, Japan; 3 Advanced Low Carbon Technology Research and Development Program, Japan Science and Technology Agency, K's Gobancho, 7 Gobancho, Chiyoda-ku, Tokyo 102-0076, Japan

## Abstract

Recently, microbe-plant interactions at the above-ground parts have attracted great attention. Here we describe nitrogen metabolism and regulation of autophagy in the methylotrophic yeast *Candida boidinii*, proliferating and surviving on the leaves of *Arabidopsis thaliana.* After quantitative analyses of yeast growth on the leaves of *A. thaliana* with the wild-type and several mutant yeast strains, we showed that on young leaves, nitrate reductase (Ynr1) was necessary for yeast proliferation, and the yeast utilized nitrate as nitrogen source. On the other hand, a newly developed methylamine sensor revealed appearance of methylamine on older leaves, and methylamine metabolism was induced in *C. boidinii*, and Ynr1 was subjected to degradation. Biochemical and microscopic analysis of Ynr1 *in vitro* during a shift of nitrogen source from nitrate to methylamine revealed that Ynr1 was transported to the vacuole being the cargo for biosynthetic cytoplasm-to-vacuole targeting (Cvt) pathway, and degraded. Our results reveal changes in the nitrogen source composition for phyllospheric yeasts during plant aging, and subsequent adaptation of the yeasts to this environmental change mediated by regulation of autophagy.

Plant leaves cover a large area of global land surface, approximately 10^9^ square kilometer^1^. Every leaf provides a habitat for colonization of microbes^2^,^3^. Many microbes form spores, either to survive under nutrient-limited conditions or to differentiate from spores into appressoria prior to invasion of host plants. Asporogenous microbes also inhabit and survive for long periods on leaf surfaces over the entire lifespan of a plant, i.e., during the growth from seed to adulthood, followed by aging and death^4^. Upon death of the host plant, microbes on the plant body return to the soil, where the plant biomass is degraded by soil microbes, recycling the nutrients for use by the next generation. Some microbes found in the phyllosphere (the above-ground portions of plants used as microbial habitats) emerge from the soil during plant growth^5^. Despite its potential importance, information regarding microbe-plant interactions and microbial physiology, in the context of the whole plant lifespan, is very limited. Consequently, the actual nutrient sources used by microbes on the above parts of plant surfaces remain mostly unknown.

Methylotrophs are microbes that can utilize reduced one-carbon (C1) compounds, e.g., methane, methanol, or methylamine, as the sole carbon and energy source. Because methanol is abundant in the phyllosphere^6^, methylotrophs are ubiquitous on plant leaf surfaces and dominate the phyllosphere population^7^. Although the associations and symbiotic relationship between plants and methylotrophic bacteria are well documented^8^,^9^,^10^, methylotrophic yeasts have not been extensively studied. Recently, we discovered that the plant-residing asporogenous methylotrophic yeast *Candida boidinii* can proliferate on growing plant leaves, assimilating methanol for its growth and survival^6^.

So far, most researchers have focused on microbe-plant interaction at underground part, and investigated microbial nitrogen metabolism, such as nitrogen fixation, nitrification, and denitrification^11^,^12^. On the other hand, above-ground part of plants, especially in the phyllsopheric environment, which is assumed to be directly affected by plant daily cycle, e.g., photosynthetic metabolism, it remains unknown which of these nitrogen compounds are utilized by microbes, although proteomic analysis and growth test analysis using growing plant tissues showed several possible nitrogen sources for microbes^13^,^14^. *C. boidinii* can utilize several nitrogen sources *in vitro*, e.g., ammonium, nitrate, and methylamine. Based on our in-house analysis of the *C. boidinii* genome analysis, we speculate that this yeast uses nitrate reductase (Ynr1) to reduce nitrate to nitrite, which is subsequently reduced to ammonium. Methylamine, on the other hand, is converted to ammonium by peroxisomal amine oxidase (Amo1) (Fig. 1a). Generally in yeasts, ammonium assimilation is catalyzed by ATP-dependent glutamine synthase, yielding glutamine from α-ketoglutarate and ammonium. Glutamine serves as a general acceptor for amino groups from amino acids and other nitrogen compounds^15^, and *C. boidinii* is thought to have a similar pathway for ammonium assimilation.

Because yeast methanol metabolism requires peroxisome biogenesis, peroxisomes are induced when cells are grown *in vitro* on methanol as the carbon source^16^,^17^. A shift to medium containing other carbon sources, or to nutrient-limited conditions, provokes degradation of peroxisomes via pexophagy, a type of selective autophagy^18^. On young plant leaves, methanol concentration exhibits a daily dynamic oscillation cycle (high during the dark period, low in the light period), and peroxisome biogenesis and pexophagy are dynamically regulated in response to the methanol cycle^6^. In a previous report, we showed that pexophagy plays crucial roles in the proliferation of *C. boidinii* on plant leaves^6^. Other lines of evidence from fungal plant pathogens indicate that pexophagy is required for invasion of fungal cells into host plants^19^.

Autophagy is a well-characterized catabolic pathway responsible for degradation of superfluous or dysfunctional cellular components^20^. This process contributes to intracellular remodeling, as well as removal of aggregates and damaged organelles. The most prevalent form of autophagy is starvation-induced macroautophagy, a nonselective system for bulk degradation of cytoplasmic components^21^. During this process, subcellular substrates are sequestered within double-membrane-bound structures called autophagosomes. These structures fuse with vacuoles, leading to the degradation or recycling of their cargo^21^.

In contrast to the bulk degradation system of macroautophagy, there also exist selective autophagic pathways, in which cytoplasmic cargoes are selectively recognized and transported to the vacuole for degradation^22^. One such selective pathway is pexophagy, described above, which is responsible for degradation of excess peroxisomes. Additional types of selective autophagy correspond to other organelles: mitophagy for mitochondria, lipophagy for lipid droplets, ribophagy for ribosomes, and ER-phagy for the endoplasmic reticulum^23^,^24^,^25^,^26^. In addition, yeasts have a constitutive biosynthetic pathway, the cytoplasm-to-vacuole targeting (Cvt) pathway, which is responsible for the transport of several vacuolar enzymes including aminopeptidase Ι (ApeΙ), aspartyl aminopeptidase (Ape4), and alpha mannosidase Ι (AmsΙ)^27^,^28^,^29^. ApeΙ, which is synthesized as a precursor ApeΙ (pre-ApeΙ), forms a large Cvt complex that is sequestered by a double-membrane structure, forming a Cvt vesicle. And after its delivery to the vacuole, pre-ApeΙ is matured by removal of the propeptide^30^.

In this study, we investigated the nitrogen-utilization pathway of the methylotrophic yeast *C. boidinii* on plant leaves. Ynr1 was necessary for the proliferation of this yeast on young leaves. On older leaves, however, methylamine played a more important role as nitrogen source, and Ynr1 was transported to the vacuole and degraded*.* Analyses of the dynamics of Ynr1 after the nitrogen-source switch revealed that Ynr1 protein is transported to the vacuole for degradation via the autophagic vesicles of biosynthetic Cvt pathway. These results elucidate yeast nitrogen metabolism and the regulation of autophagy on plant leaves in response to the life stages of the host plant.

## Results

### *C. boidinii* utilizes nitrate on growing *A. thaliana* leaves

**Figure 1:**
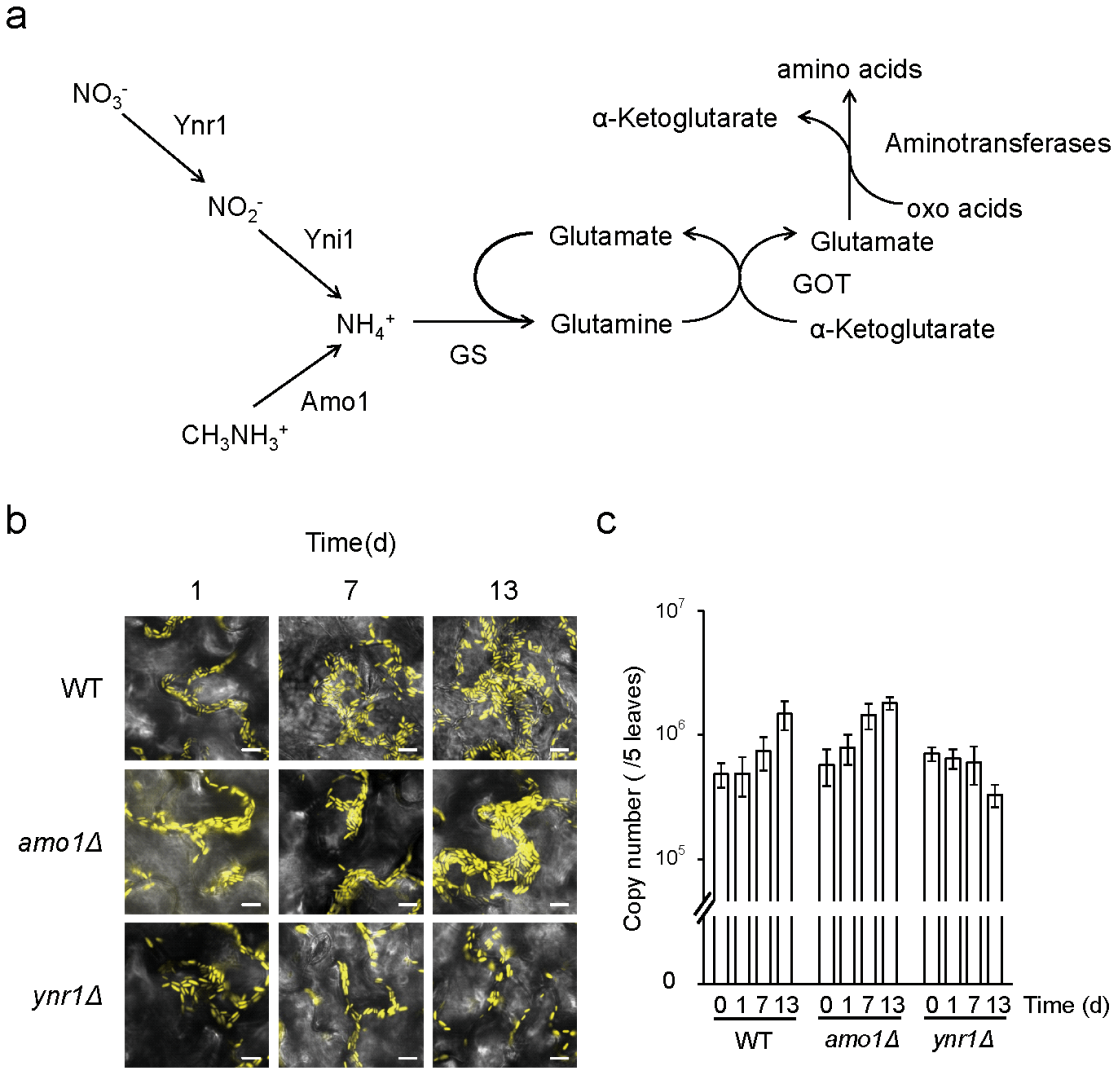
Ynr1 is necessary for yeast proliferation on *A. thaliana* leaves. (a) Nitrogen utilization pathway in the methylotrophic yeast *C. boidinii*. Ynr1, nitrate reductase; Yni1, nitrite reductase; Amo1, amine oxidase; GS: glutamine synthetase; GOT, glutamine oxoglutarate aminotransferase. (b) Confocal microscope images of Venus-labeled wild-type, *amo1Δ*, and *ynr1Δ* strain on growing *A. thaliana* leaves. *C. boidinii* cells were spotted on young *Arabidopsis* leaves (2–3 weeks after germination) and observed on the indicated day. Bar, 10 µm. (c) Quantitation of *C. boidinii* cell populations on the *A. thaliana* leaves by qPCR method. The leaves onto which the *C. boidinii* strains were inoculated were retrieved at the indicated time points, and were subjected to qPCR that amplified *VENUS* gene integrated in the *C. boidinii* genome. The Error bars show standard deviations of triplicate measurements.

Homology searches of the *C. boidinii* genome database revealed the genes encoding enzymes involved in nitrate- and methylamine-utilization pathways predicted to yield ammonium. The genes encoding the initial steps for these pathways are *YNR1*, encoding the nitrate reductase Ynr1, and *AMO1*, encoding the amine oxidase Amo1 (Fig. 1a). These genes were first assigned based on the alignment with orthologous enzymes from the methylotrophic yeast *Hansenula polymorpha* (Supplementary Fig. 1a,b). In order to reveal the functions of *YNR1* and *AMO1* in *C. boidinii*, these genes were disrupted by replacing the appropriate ORF with the Zeocin^TM^ resistance gene as a selective marker. The *ynr1Δ* and *amo1Δ* strains could not utilize nitrate and methylamine, respectively, as the nitrogen source (Supplementary Fig. 2a,b,c). Furthermore, nitrate reductase and amine oxidase activities were lost in the *ynr1Δ* and *amo1Δ* strains, respectively (Supplementary Table 1), confirming that these genes encode proteins with the corresponding activities.

A previous study established a technique for evaluating proliferation of *C. boidinii* on plant leaves using a combination of qPCR and fluorescent microscopy^6^. In this study, we used that technique to compare the growth of the *ynr1Δ* and *amo1Δ* strains on young *A. thaliana* leaves (2–3 weeks after germination) with that of a corresponding strain expressing Venus under the control of the constitutive *ACT1* promoter. In the wild-type and *amo1Δ* strains, concomitant with increases in the cell numbers observed under fluorescence microscopy (Fig. 1b), 4–8 fold increases in the copy numbers of *VENUS* gene integrated in the *C. boidinii* genome were detected after 13 days of inoculation, indicating proliferation of the strains on the leaves. On the other hand, such proliferation was not observed for the *ynr1Δ* strain (Fig. 1b, c). These results indicated that *YNR1* is necessary for *C. boidinii* proliferation on growing young *A. thaliana* leaves, and that *C. boidinii* can utilize nitrate as a nitrogen source.

### *YNR1*, but not *AMO1*, is expressed on young leaves

Next, we examined the regulation of *YNR1* and *AMO1* expression*,* as well as the activities of the corresponding enzymes, in response to various nitrogen sources. *YNR1* and *AMO1* were specifically induced by nitrate and methylamine, respectively, at both the enzyme-activity (Supplementary Table 2) and transcript levels (Supplementary Table 3).

**Figure 2:**
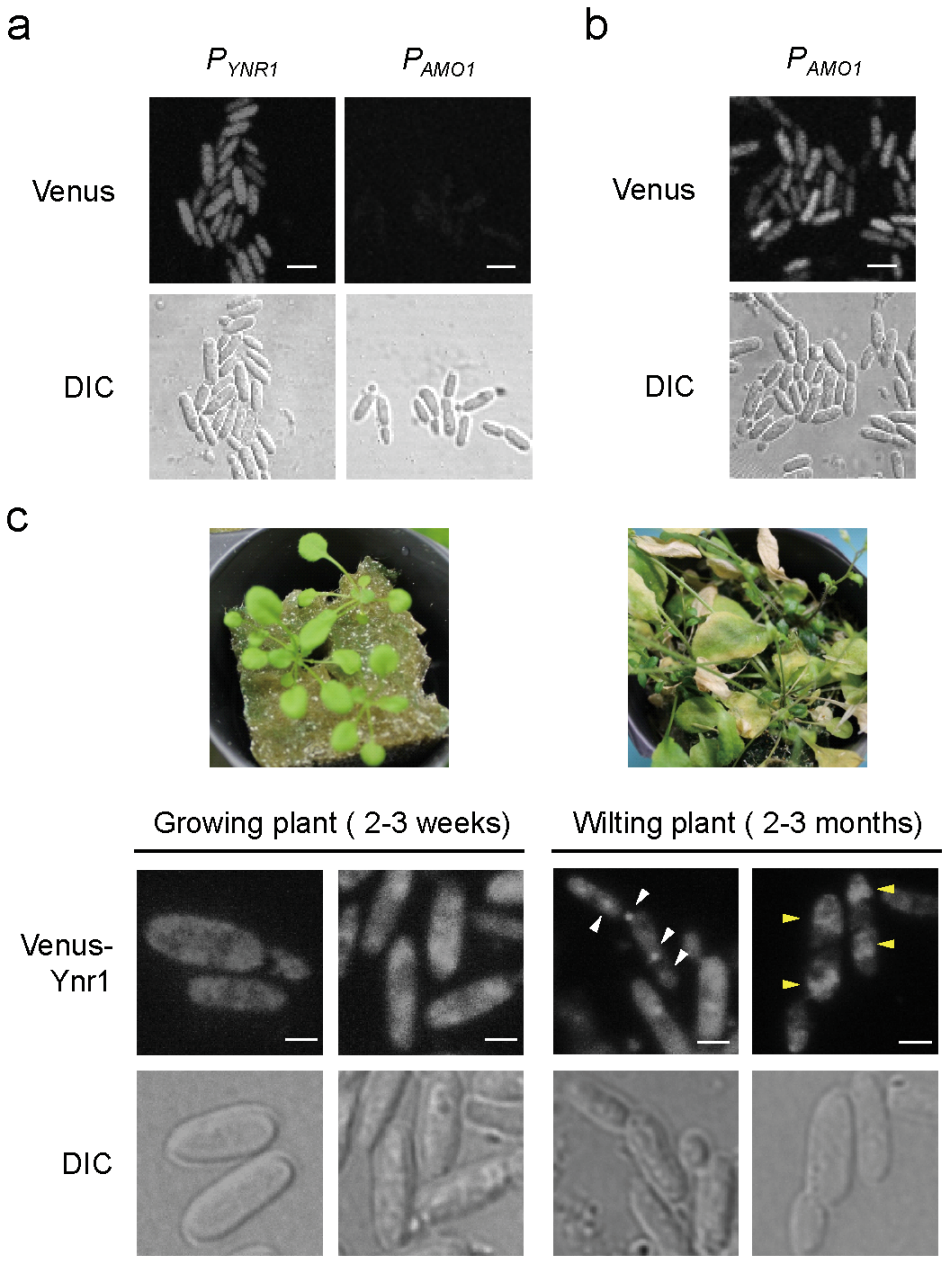
Expression of *YNR1* and *AMO1* on the leaves of *A. thaliana*. (a) Microscopic images of *C. boidinii* inoculated onto the surface of a young leaf. Venus fluorescent protein was expressed under the control of the *YNR1* or *AMO1* promoter. DIC represents the image from differential interference contrast microscopy. Bar, 5 µm. (b) Microscopic images of *C. boidinii* inoculated onto the surface of a wilting leaf. Venus fluorescent protein was expressed under the control of the *AMO1* promoter. Bar, 5 µm. (c) Intracellular localization of Venus-Ynr1 on plant leaves. Venus-Ynr1 fusion protein was expressed under the control of the native *YNR1* promoter in cells living on growing or wilting leaves of *A. thaliana* shown in the upper images. White arrowheads show dot-like structures of Venus-Ynr1, and yellow arrowheads show the diffuse Venus fluorescence within the vacuole. Bar, 2 µm.

Next, we constructed strains expressing Venus under the control of the *YNR1* promoter (strain PYNR) or the *AMO1* promoter (strain PAMO), and then examined their promoter activities on young plant leaves. At 4 h of incubation after spotting onto the upper side of growing young leaves, the cells were observed under a fluorescent microscope. Strain PYNR exhibited clear cytosolic fluorescence, whereas strain PAMO did not (Fig. 2a). Thus, *YNR1* was expressed on young leaves of *A. thaliana*, whereas *AMO1* was not. Therefore, we hypothesized that *YNR1* expression is induced by nitrate present on plant leaves.

The genes for methanol utilization exhibit a daily periodicity in their expression, reflecting the oscillation in methanol concentration: high expression during the dark period and low levels during the light period. For example, the transcript level of *DAS1*, which encodes peroxisomal dihydroxyacetone synthase, is ~3-fold higher in the dark period than the light period^6^. Therefore, we investigated whether the transcript level of *YNR1* changes during the daily cycle. To this end, the total RNA was extracted from *C. boidinii* proliferating on leaf surfaces, and subjected to qRT-PCR analysis. The transcript level of *YNR1* increased 2.8-fold, peaking at 8 hh (4 h after the start of light period), decreasing gradually to its minimum at 4 hh (8 h after the start of dark period) (Supplementary Fig. 3a). These findings indicate that *YNR1* expression also fluctuates on young plant leaves during the daily cycle.

### *AMO1* expression and alteration of Venus-Ynr1 localization on aged leaves

During the plant life cycle (growth, maturation, wilting, and death), microbes in the phyllosphere must adapt to host aging–dependent changes in the leaf environment. On young growing leaves, methanol concentration in the phyllosphere oscillates in a daily cycle. This oscillation disappears in wilting leaves, and the concentration steadily rises as wilting progresses and the leaf dies. On old leaves, yeasts are unable to proliferate; instead, they survive by using their highly developed peroxisomes as protein-storage organelles^6^.

Using strain PAMO, we investigated whether *AMO1* was expressed on wilting plant leaves (2–3 months after germination). Cytosolic fluorescence was detectable on wilting leaves (Fig. 2b), suggesting that methylamine metabolism is induced in *C. boidinii* on an aged or dead host plant. Using a standard curve of fluorescence intensity, based on agar plates containing various concentrations of methylamine (Supplementary Fig. 4), we determined that methylamine concentration at the phyllosphere was on average 4.78 × 10^−3^ mM.

To determine the localization of Ynr1 on aged plants, we constructed a strain expressing a Venus-Ynr1 fusion protein under the control of the *YNR1* promoter. Expression of Venus-Ynr1 protein fully restored the growth of *ynr1*Δ strain on nitrate as a solo nitrogen source (data not shown). When Venus-Ynr1 expressing cells were inoculated on young leaves of growing plant (2–3 weeks after germination), we observed cytosolic fluorescence (Fig. 2c). On the other hand, we detected dot-like structures of Venus-Ynr1 and diffuse vacuolar fluorescence in cells inoculated onto aged leaves of wilting plant (2–3 months after germination) (Fig. 2c). These results prompted us to hypothesize that Ynr1 is subjected to autophagic degradation induced by the adaptation to the change of leaf environment.

### Ynr1 activity decreased after the shift from nitrate- to methylamine-containing medium

**Figure 3:**
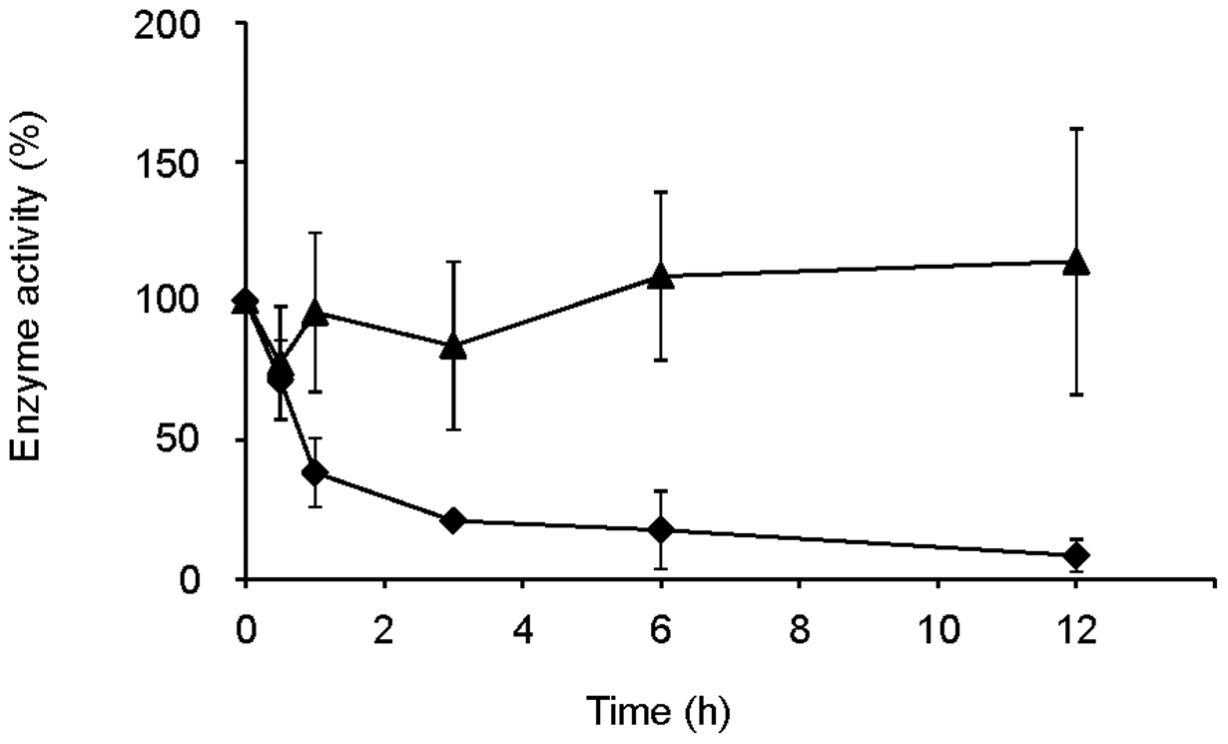
Ynr1 enzyme activity decreases rapidly after nitrogen-source shift. Symbols: ♦, 10 mM methylamine; ▴, 10 mM nitrate. Experiments were repeated three times. Enzyme activity is expressed as the relative value to each of the values measured at the time point 0 h: (♦) 73.2 and (▴) 87.9 U (µmol/mg protein). Error bars show the standard deviations of three independent experiments.

To understand the adaptation mechanism of the yeast for environmental change on leaves over the course of host plant aging, we decided to examine the dynamics of Ynr1 *in vitro*. First, we monitored the enzyme activity of Ynr1 following a shift from nitrate- to methylamine-containing liquid medium. Specifically, we grew wild-type *C. boidinii* on 10 mM nitrate as the sole nitrogen source (nitrate medium), and then transferred them to 10 mM methylamine (methylamine medium) or nitrate medium (as a control.) The enzymatic activity of Ynr1 decreased rapidly within 1 h of the shift from nitrate to methylamine medium. By contrast, transfer to nitrate medium did not significantly affect enzymatic activity (Fig. 3).

### Ynr1 is transported to the vacuole via autophagy after the shift to methylamine

Because Ynr1 enzyme activity decreased after the nitrogen-source shift, we hypothesized that Ynr1 was subjected to autophagic degradation. To elucidate the molecular basis of autophagic degradation of Ynr1 after the nitrogen-source shift, we used a strain in which Venus-Ynr1 fusion protein was expressed under the *YNR1* promoter. Once Venus-Ynr1 is delivered to the vacuole, the Venus moiety is proteolytically detached from the rest of the fusion protein, after which it is relatively stable and can be detected by immunoblot analysis^31^.

**Figure 4:**
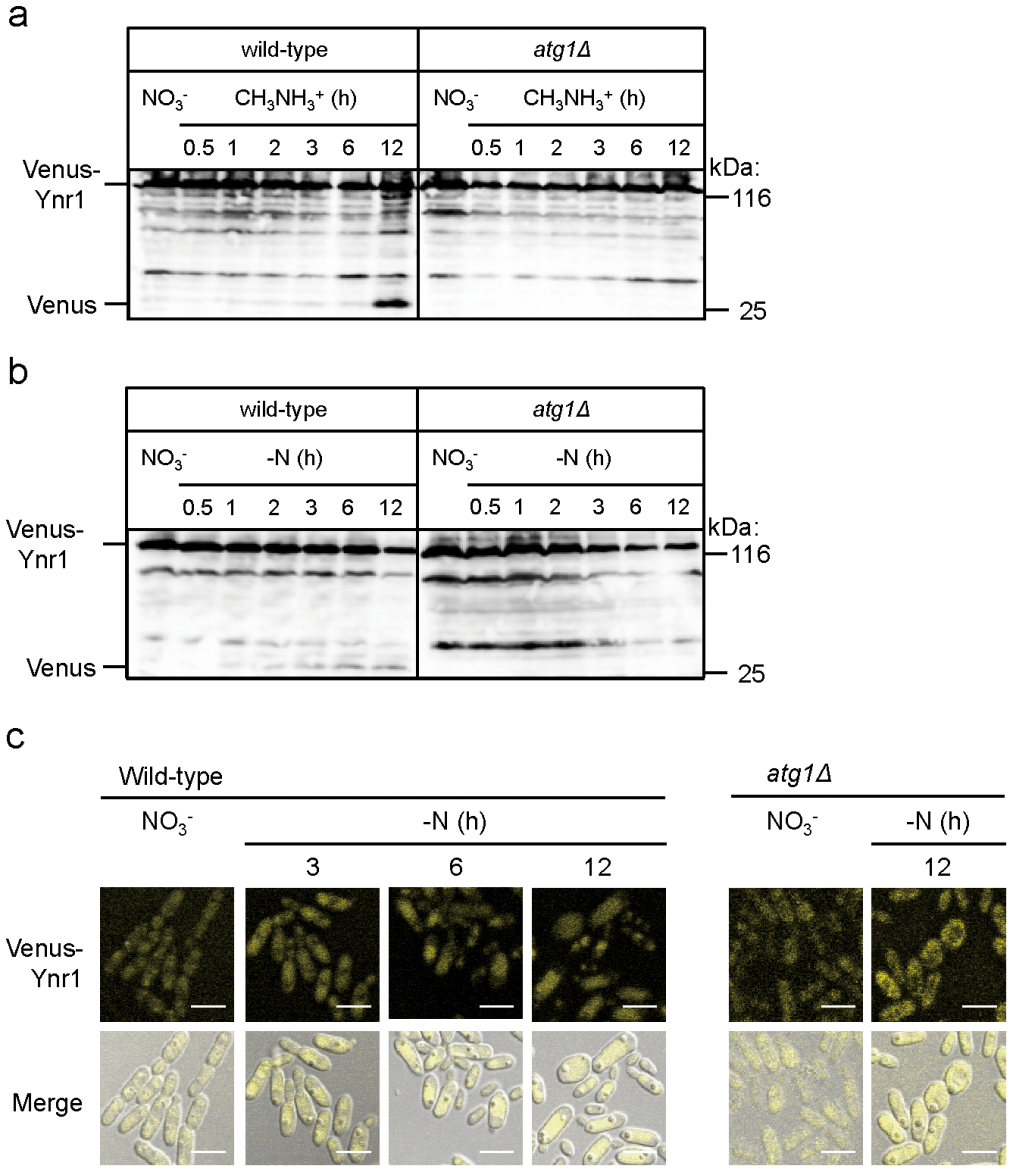
Ynr1 transport to the vacuole is defective in *atg1Δ* cells. (a) Immunoblot analysis of Venus-tagged Ynr1 in *C. boidinii* wild-type and *atg1Δ* cells transferred from nitrate to methylamine medium. (b) Immunoblot analysis of Venus-tagged Ynr1 in *C. boidinii* wild-type and *atg1Δ* cells transferred from nitrate medium to nitrogen-starvation conditions. (c) Microscopic images of Venus-Ynr1 expressed in wild-type and *atg1Δ* cells during the shift from nitrate medium to nitrogen-starvation conditions. Merged images are combined images of Venus yellow fluorescence and DIC images. Bar, 5 µm.

We cultivated wild-type yeast as well as *atg1Δ* and *atg8Δ* cells, which are impaired in all autophagic processes, on medium containing on nitrate, and then shifted the cells to methylamine medium or medium without a nitrogen source (nitrogen starvation). Twelve hours after the shift from nitrate medium to methylamine medium, the cleaved form of Venus was detected in wild-type cells, but not in *atg1Δ* cells or *atg8Δ* cells (Fig. 4a and Supplementary Fig. 5a). In a similar manner, when the cells were subjected to nitrogen-starvation conditions to induce bulk autophagy, the cleaved form of Venus was detected in the wild-type strain but not the *atg1Δ* strain (Fig. 4b).

Interestingly, the band intensities of the cleaved form of Venus, detected after the medium transfer from nitrate to methylamine, were more intense than those detected after the shift to nitrogen-starvation conditions (Fig. 4b). These results suggest that Ynr1 is more effectively subjected to degradation after a nitrogen-source shift from nitrate to methylamine than under conditions that induce bulk autophagy.

We also used fluorescence microscopy to monitor the fate of Venus-Ynr1 following the medium shift. After the transition from nitrate to nitrogen-starvation medium, Venus fluorescence was detected within the vacuole in wild-type cells, but remained in the cytosol in *atg1Δ* cells (Fig. 4c). These morphological results were also consistent with the biochemical analysis, which indicated that Venus-Ynr1 is transported to the vacuole via autophagy after the shift of nitrogen source.

### Transport of Ynr1 to the vacuole requires the selective autophagy factor Atg11

**Figure 5:**
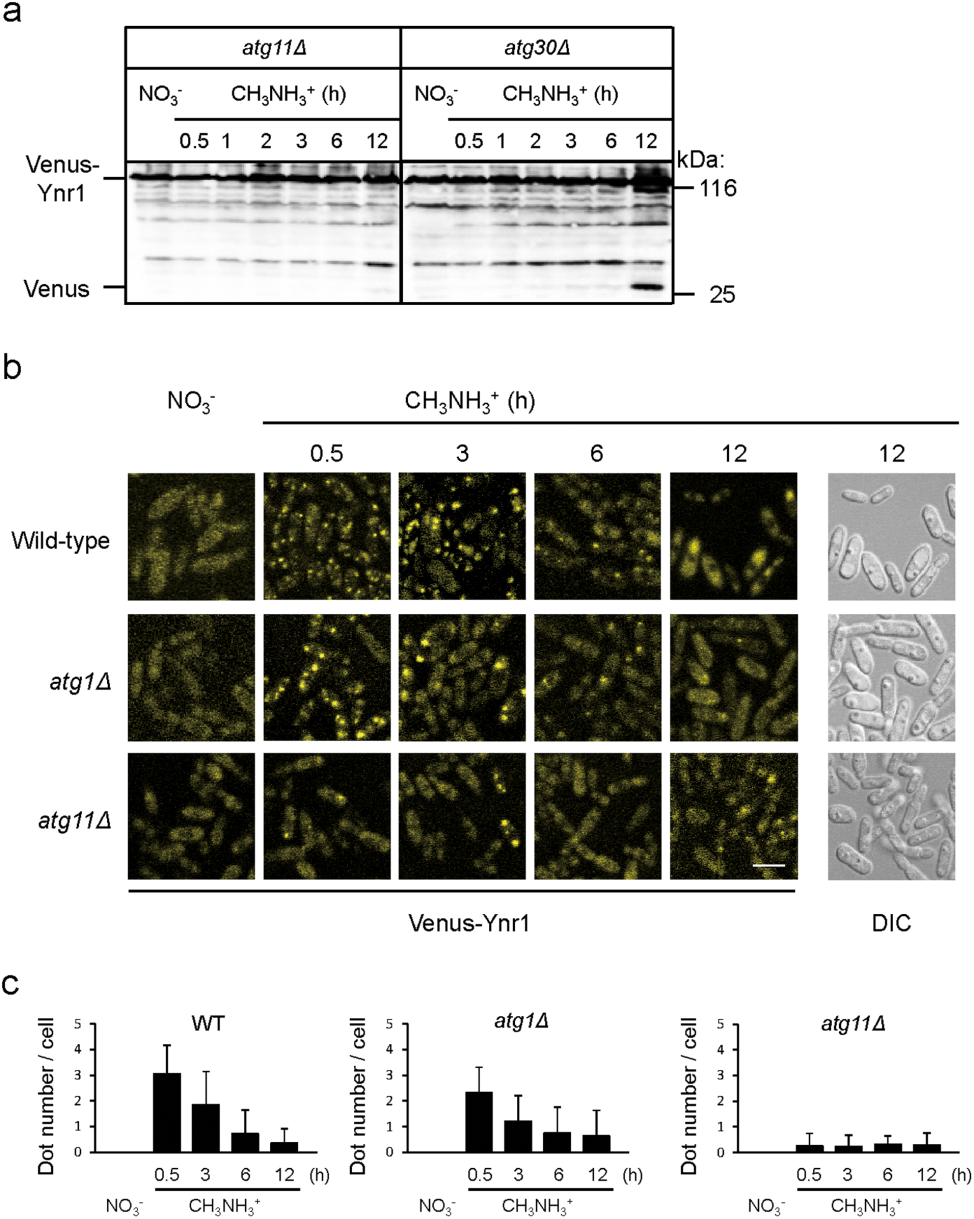
Ynr1 transport to the vacuole is dependent on the selective autophagy factor Atg11. (a) Immunoblot analysis of Venus-tagged Ynr1 in *atg11Δ* and *atg30Δ* strains transferred from nitrate to methylamine medium. (b) Fluorescent images of Venus-Ynr1–expressing *C. boidinii* wild-type, *atg1Δ*, and *atg11Δ* cells during the nitrogen-source shift from nitrate to methylamine. Bar, 5 µm. (c) Quantitation of the number of Venus-Ynr1 puncta per cell, estimated from analysis of the fluorescent images shown in (b). For each sample, a minimum of 50 cells were analyzed. Error bars show standard deviations.

Atg11 is required for selective autophagy pathways, including the Cvt pathway, mitophagy, and pexophagy. It functions as a scaffold protein for recruitment of cargo to the phagophore assembly site (PAS) for packaging into vesicles^22^. We investigated whether Ynr1 is transported to the vacuole in an Atg11-dependent manner. In *atg11Δ* cells, the band intensity of the cleaved form of Venus was clearly weaker than that in wild-type cells after the medium shift from nitrate to methylamine (Fig. 5a).

Amo1 is a peroxisomal amine oxidase, which has a peroxisomal targeting signal type 2 (PTS2) motif with the consensus sequence of (R,K)-(L,V,I)-X5-(H,Q)-(L,A,F). During adaptation to methylamine medium in the methylotrophic yeast *Hansenula polymorpha*^32^, Amo1 was induced and transported to the peroxisomes. Atg11 is also necessary for pexophagy^33^. Therefore, we asked whether Atg11-dependent transport of Venus-Ynr1 was independent of pexophagy. In the methylotrophic yeast *Pichia pastoris*, Atg30 is required for pexophagy, but not for the Cvt pathway or mitophagy^34^*.* Twelve hours after the medium shift, we detected the cleaved form of Venus in *atg30Δ* cells, as well as in wild-type cells, indicating that Ynr1 transport to the vacuole is independent of pexophagy (Fig. 5a). Ynr1 was also degraded in the *atg17Δ* strain, which is impaired in non-selective bulk autophagy (Supplementary Fig. 5b).

We also observed the localization of Venus-Ynr1 by fluorescence microscopy. When cells were cultured on nitrate medium, Venus-Ynr1 was found dispersed in the cytosol (Fig. 5b). In wild-type cells, 0.5 h after the shift from nitrate to methylamine medium, Venus-Ynr1 formed dots, which were detectable for further 3 h. Subsequently, the Venus fluorescence was localized inside the vacuole until 12 h after the shift. In *atg1Δ* and *atg8Δ* cells, in contrast, Venus-Ynr1 was not transported to the vacuole (Fig. 5b and Supplementary Fig. 5c). Atg11 and Atg17 serve as scaffold proteins for phagophore formation in the selective and non-selective autophagic pathways, respectively. The frequency of Venus-Ynr1 dot formation was low in *atg11Δ* cells, (Fig. 5b,c), but normal in *atg17Δ* cells (Supplementary Fig. 5c,d). Thus, trafficking of Venus-Ynr1 to the vacuole depended on selective autophagy but not on non-selective autophagy. Indeed, after the medium shift, Venus expressed in the cytosol did not exhibit vacuolar fluorescence (Supplementary Fig. 5e) indicating that starvation-induced autophagy did not occur. These biochemical and morphological data confirmed that Ynr1 is selectively transported to the vacuole in Atg11-dependent manner.

### Ynr1 is transported to the vacuole via the Cvt pathway during adaptation from nitrate to methylamine medium

**Figure 6:**
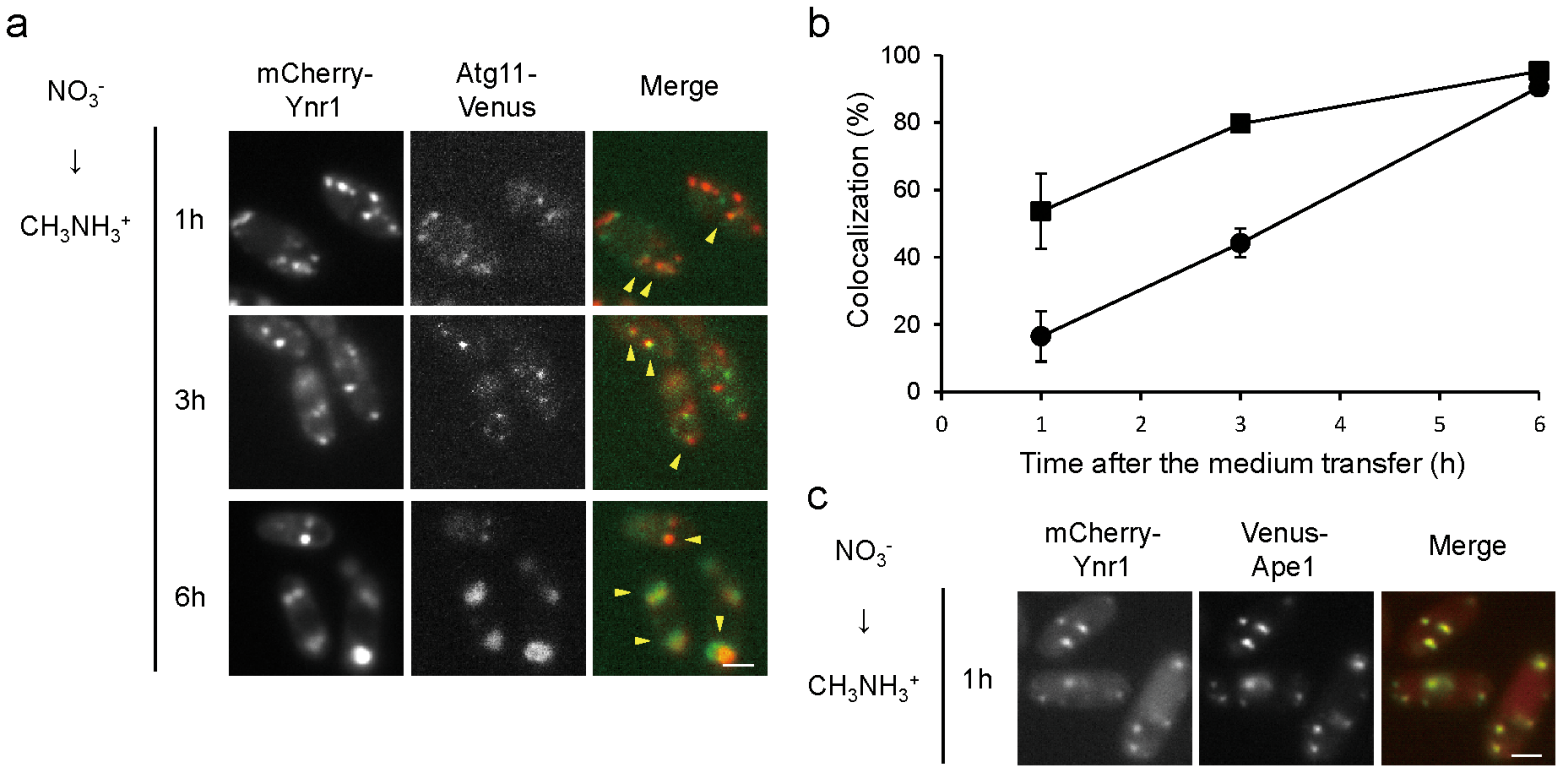
Sequestration and transportation of Ynr1 via the Cvt pathway after the shift from nitrate to methylamine. (a) Microscopic images of *C. boidinii* wild-type cells expressing Atg11-Venus and mCherry-Ynr1. The cells were subjected to the nitrogen-source shift from nitrate to methylamine. Yellow arrowheads indicate co-localization of Atg11-Venus and mCherry-Ynr1. Merged images are combined images of Venus green fluorescence and mCherry red fluorescence images. Bar, 2 µm. (b) Quantitation of co-localization of Atg11-Venus and mCherry-Ynr1 in the *C. boidinii* wild-type strain used for the microscopic imaging analysis shown in (a). Percentages represent the ratio of the number of co-localized puncta to the number of Atg11-Venus puncta or mCherry-Ynr1 puncta. Symbols: 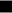, ratio of the number of co-localized puncta to the number of Atg11-Venus puncta; 
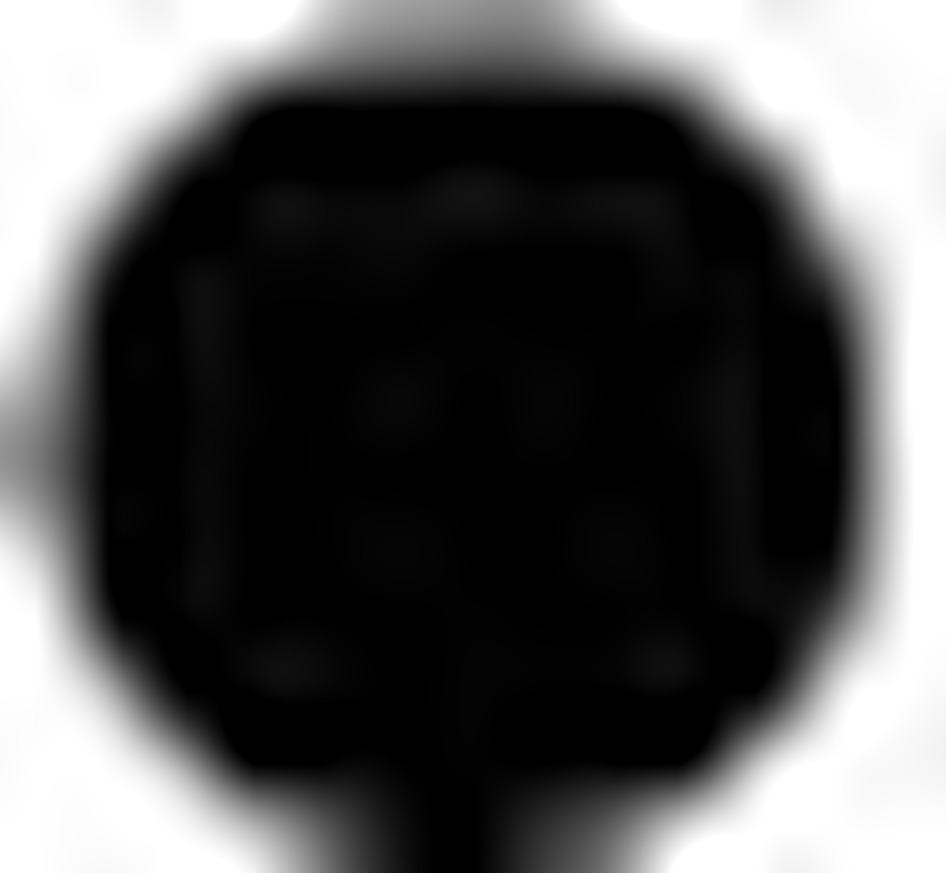
 , ratio of the number of co-localized puncta to the number of mCherry-Ynr1 puncta. For each sample, a minimum of 50 cells was analyzed. Error bars show standard deviations of dot measurements of all cells. (c) Co-localization of Venus-Ape1 and mCherry-Ynr1 in wild-type cells 1 h after the nitrogen-source shift from nitrate to methylamine. Yellow arrowheads indicate co-localized puncta. Bar, 2 µm.

We interpreted Venus-Ynr1 dot formation as a sign of sequestration into autophagosomes. To test this idea, we constructed wild-type cells expressing both mCherry-Ynr1 and Atg11-Venus, and examined the subcellular localization of both proteins. Following the shift from nitrate to methylamine, mCherry-Ynr1 and Atg11-Venus were co-localized to an extent that increased over time (Fig. 6a,b). Atg8 is an autophagosome marker that has been used previously to follow the dynamics of autophagy^35^, and co-localization of mCherry-Ynr1 and Venus-Atg8 was also observed in the wild-type strain (Supplementary Fig. 6a). These results suggested that Ynr1 is selectively recruited and sequestered into autophagic vesicles for its transport to the vacuole.

To examine the involvement of Cvt pathway in the Ynr1 transport to the vacuole, we constructed wild-type cells expressing both mCherry-Ynr1 and Venus-Ape1. When the cells were shifted from nitrate to methylamine medium, mCherry-Ynr1 often localized with Venus-Ape1 (Fig. 6c). We also observed co-localization of mCherry-Ynr1 and Ape4-Venus, another member of the Cvt complex^28^ (Supplementary Fig. 6b). These results demonstrate that Ynr1 forms dots associated with the Cvt complex, is sequestered into Cvt vesicles, and is ultimately delivered to the vacuole. These data indicated that Ynr1 forms a complex for its degradation with other vacuolar enzymes for biosynthesis.

### Ynr1 is degraded via the Cvt pathway on wilting plant leaves

**Figure 7:**
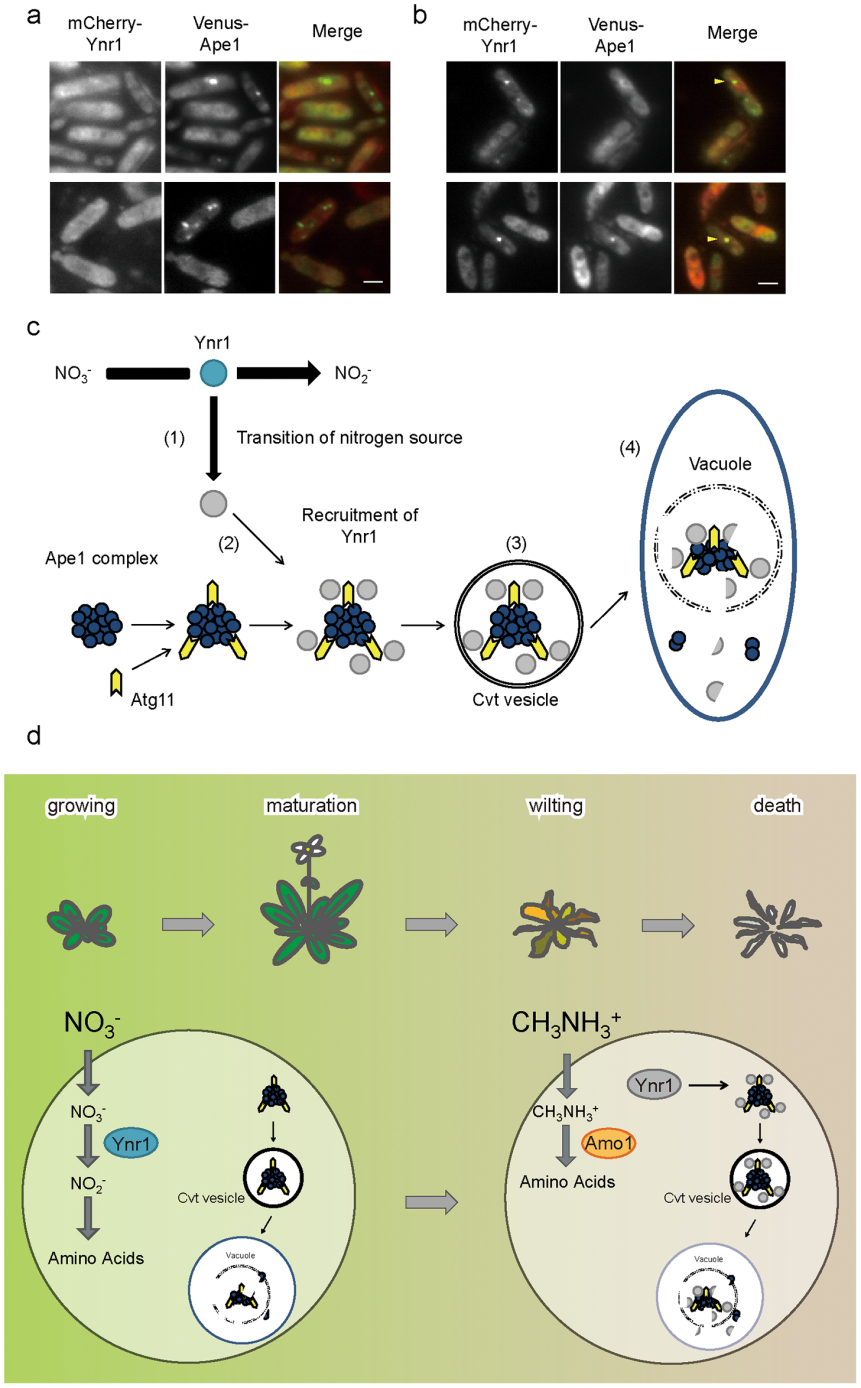
Co-localization of mCherry-Ynr1 and Venus-Ape1 in *C. boidinii* cells inoculated on wilting plant leaves. (a) and (b) Microscopic images of *C. boidinii* cells expressing mCherry-Ynr1 and Venus-Ape1 on (a) growing or (b) wilting *A. thaliana* leaves. Yellow arrowheads indicate co-localization of Atg11-Venus and mCherry-Ynr1. Merged images are combined images of Venus green fluorescence and mCherry red fluorescence images. Bar, 2 µm. (c) Ynr1 turnover on plant leaves via the Cvt pathway. On growing plant leaves, *C. boidinii* utilizes nitrate as the nitrogen source, and active Ynr1 (blue) catalyzes reduction of nitrate to nitrite. The transcriptional level of *YNR1* is adjusted in accordance with the plant's daily light–dark cycle. As the plant gets older, methylamine (and other compounds) leaks from the plant surface and can be used as a nitrogen source by *C. boidinii*. (1). At this stage, inactive Ynr1 (gray) is recruited to the Ape1 complex (2) in atg11-dependent manner. After sequestration of Ynr1 inside the Cvt vesicle (3), the vesicle fuses with the vacuole, releasing the Cvt body into the vacuolar lumen (4). Released Ynr1 is subjected to degradation. (d) Nitrogen metabolism and regulation of autophagy in the methylotrophic yeast in the phyllosphere. On growing leaves, nitrate can be utilized as nitrogen source. On the other hand, on wilting leaves, methylamine is utilized by *C. boidinii* and Ynr1 is inactivated, incorporated into the Cvt vesicle with other vacuolar hydrolases, and degraded.

Finally, we monitored the localization of mCherry-Ynr1 and Venus-Ape1 in *C. boidinii* cells grown on plant leaves. As shown in Fig. 7a,b, Venus-Ape1 formed dots in cells grown on both young and wilting plant leaves. On the other hand, dot-like structures of mCherry-Ynr1 were observed only on wilting plant leaves (Fig. 7b), although the number and size of these dots were smaller than those detected in cells grown in methylamine-containing liquid media. These results support the idea that the active form of Ynr1 catalyzes reduction of nitrate to nitrite in the cytosol of cells growing on young plant leaves, whereas on old leaves Ynr1 is transported to the vacuole as a new cargo of the biosynthetic Cvt vesicles, and degraded (Fig. 7c).

## Discussion

Asporogenous yeasts that inhabit plant surfaces are speculated to evolve other strategies than forming spores, in order to survive environmental changes in their ecological niche. Here we show that nitrate was used by the yeast *C. boidinii* on younger leaves while methylamine became more important as nitrogen source on older leaves, and demonstrate that nitrate reductase Ynr1, essential for yeast proliferation on young leaves, was transported to and degraded in the vacuole mediated by an Atg11-dependent selective pathway similar to the Cvt pathway on aged leaves. The Cvt pathway is a constitutive biosynthetic pathway, and Ape1 transport to vacuole was indeed suggested on young leaves (Fig. 7a,b). On aged leaves, Ynr1 became a cargo member of Cvt vesicles, and was degraded in the vacuole (Fig. 2c and Fig. 7b). Because Amo1 is transported to peroxisomes and catalyzes methylamine metabolism on aged leaves, it is logical for Ynr1 degradation to be independent of pexophagy.

Regulation of autophagy in *C. boidinii* plays a critical role in the daily environmental adaptation during growth on young leaves^6^. Under such conditions, some type of autophagy (e.g., the Cvt pathway) was constitutively activated, as judged by cleavage of Venus-Atg8 and lipidation of Atg8, but Ynr1 escaped degradation. By contrast, both peroxisome proliferation and pexophagy are regulated by the daily cycle of methanol concentration, the main carbon source for this organism on plant leaves. Pexophagy occurs only during the light period when the methanol concentration is low^6^, and this specific degradation of peroxisomes, mediated by Atg30, is necessary for proliferation of this yeast on young growing plant leaves. In addition to the plant daily light-dark cycle, we herein showed the importance of regulation of autophagy in order to adjust a dynamically changing environment in response to aging of host plant. Ynr1, which functions on young leaves, is not degraded via autophagy on young plant leaves. However, on wilting plant leaves, it becomes a cargo for the constitutive Cvt pathway, which has shown to occur on plant. And also, our results propose a new role of Cvt pathway for protein degradation, that has been believed to be a biosynthetic pathway. Together with the results of these recent studies, our findings reveal that selective autophagy is strictly controlled in a sophisticated manner. To the best of our knowledge, these findings represent the first description on how eukaryotic microbes surviving on plant surfaces adapt to the environment by regulating multiple autophagic pathways in a sophisticated and strict manner not only during the daily cycle but also during the plant lifespan. Such regulation of autophagy was suggested to exist also in a plant pathogenic fungi during differentiation of appressorium on plant leaf^19^.

We also investigated the role of autophagy on Ynr1 activity *in vitro* during a nitrogen-source shift from nitrate to methylamine. Following the nitrogen-source shift, Ynr1 was rapidly inactivated and decreased to ~20% of its enzymatic activity after 6 h. On the other hand, we detected the cleaved form of Venus only 12 h after the medium transfer, indicating that Ynr1 was inactivated prior to its transport to the vacuole for degradation. The enzyme activity decreased at similar rates in the *atg1Δ* and *atg11Δ* strains. Although activation of Ynr1 in *H. polymorpha* has been studied at the level of expression^36^,^37^, inactivation of yeast Ynr1 has not been reported previously. On the other hand, nitrate reductases from higher plants contain a well-conserved serine residue within the hinge 1 region that is phosphorylated in response to light or CO_2_^38^. We could neither find a conserved region in Ynr1 nor detect phosphorylation of Ynr1 during the process of adaptation to methylamine. We speculate that inactivated Ynr1 can be distinguished from its active form, and can therefore be recognized by some autophagic receptor protein and thereby recruited to Cvt vesicles for degradation. Atg19, a receptor protein of the Ape Ι complex, is not present in *C. boidinii*. Thus, the detailed mechanisms underlying inactivation of Ynr1, and specific recruitment of the inactivated enzyme to Cvt vesicles, remain to be elucidated.

By quantitative growth analysis, we showed that the *C. boidinii ynr1Δ* strain could not proliferate on young leaves. Therefore, nitrate sufficient to support yeast proliferation is present on young leaves. It has been widely assumed that nitrate is an abundant compound in many plants, and that plants can store excess nitrate in their tissues, including leaves^39^,^40^,^41^. Furthermore, nitrate assimilation was recently shown to be important in colonization of plant leaves by an aerobic Gram-negative and asporogenous bacterium^14^. Although all of these observations suggested that nitrate available for microbes is present on leaves, our observation of the impaired growth of the *C. boidinii ynr1Δ* strain on plant leaves provides the first demonstration that microbes utilize nitrate on young plant leaves.

Ynr1 expression oscillated during the daily cycle on young leaf, in a cycle similar to the daily oscillations of methanol, methanol-inducible genes, and peroxisome dynamics (Supplementary Fig. 3). Although we could not estimate the nitrate concentration in the phyllosphere in *C. boidinii* strain PYNR, which expressed Venus from the *YNR1* promoter, previous reports have indicated that the nitrate concentration in plant leaves is several times higher in the light than in the dark^42^,^43^. Furthermore, nitrate reductase activity in leaves is higher in the light period, and it is rapidly inactivated in the dark^44^. Therefore, it is likely that the daily oscillation of *YNR1* expression reflected the daily fluctuation of nitrate concentration in the phyllosphere.

Another important finding of this study is that the importance of particular nitrogen sources for microbes in the phyllosphere changes during the plant life cycle, and microbes must also adapt to this change. On aged leaves, *C. boidinii* cells express peroxisomal Amo1 and utilize methylamine (and possibly other unidentified nitrogen compounds) that leak from the plant cells. At this stage, Ynr1 is transported to the vacuole for degradation. When we used a *C. boidinii* cell sensor expressing the *AMO1* promoter–driven Venus to assess the local methylamine concentration on *A. thaliana* leaves, we did not detect methylamine on growing young leaves (Fig. 2a). By contrast, on aged plants, methylamine was estimated to be present at a concentration of 4.78 × 10^−3^ mM. Intracellular nitrogen compounds leak from the aged plant cells, resulting in a dramatic change in the importance of each nitrogen source for phyllospheric microbes. These changes will affect the population and activity of microbes on alive and dead plant surfaces, mediating environmental element circulation by decomposing dead organisms.

To further understand the adaptation mechanisms of the yeast to the change in leaf environment during plant lifespan, we set up and conducted *in vitro* culture experiments, and found that the change of nitrogen source from nitrate to methylamine induced degradation of Ynr1 via selective autophagy. While we could observe induction of *AMO1* expression and the appearance of Ynr1 punctates on aged leaves, these might not be due to simple change of nitrogen source from nitrate to methylamine rather due to complex environmental changes in the phyllosphere.

In conclusion, we elucidated the influence of nitrogen metabolism on the regulation of autophagy in *C. boidinii* on both growing and wilting leaves of *A. thaliana* (Fig. 7d). On growing leaves, *C. boidinii* utilizes nitrate and adjusts the expression levels of *YNR1* in accordance with daily oscillation of nitrate concentration. During the light period, while pexophagy is active on young leaves, Ynr1 escapes from autophagic degradation. In contrast, on wilting leaves, the yeast responds to the change of leaf environment by expressing peroxisomal Amo1 and degrading cytosolic Ynr1 via the Cvt pathway. Overall, we showed that the composition of available nitrogen sources for phyllospheric yeasts changes during plant lifespan. Moreover, our study has shed light on a new role of Cvt pathway for protein degradation together with the importance of regulation of autophagy in eukaryotic microbes as the survival strategy on the plant surfaces. These will improve our knowledge and concept of how microbes adjust to changing of environmental conditions and how they survive in nature.

## Methods

### Yeast strains, vectors, and plasmids

The wild-type, *atg1Δ*, *atg8Δ* and *atg30Δ* *C. boidinii* strains were described previously^6^. The *ynr1Δ* and *amo1Δ* strains were constructed by replacing the appropriate ORF of *C. boidinii* strain TK62 (*ura3*^45^) with the Zeocin^TM^ resistance gene as a selective marker^46^, using the modified lithium acetate method^47^. The *atg11Δ* and *atg17Δ* *C. boidinii* strains were constructed in the same way with the primer sets Fw-ATG11u-*Pst*I/Rv-ATG11u-*Not*I and Fw-ATG17u-*Eco*RI/Rv-ATG17u-*Bam*HI for their upstream regions, and Fw-ATG11d-*Xho*I/Rv-ATG11d-*Pst*I and Fw-ATG17d-*Cla*I/Rv-ATG17d-*Eco*RI for their downstream regions, respectively. The vector pACT1-Venus, encoding Venus under the control of the *ACT1* promoter^6^, was transformed into the *ynr1Δ* and *amo1Δ* strains. The strain expressing Venus-Ynr1 fusion protein under the control of the *YNR1* promoter was used for fluorescence microscopy and immunoblot analysis. The plasmid pEX-Venus-Atg8, encoding Venus-Atg8 under the control of the *ATG8* promoter^6^, the plasmid pACT1-Venus-Ape1, encoding Venus-Ape1 under the control of the *ACT1* promoter, and the plasmid pACT1-Ape4-Venus, encoding Ape4-Venus under the control of the *ACT1* promoter were also transformed into the wild-type strain. The PYNR strain and the PAMO strain which express Venus under the *YNR1* promoter and the *AMO1* promoter, respectively, were used for expression tests on plant leaves. The nucleotide sequences of *CbYNR1*, *CbAMO1*, *CbATG11*, *CbATG17*, *CbAPE1*, and *CbAPE4*, were deposited in the DDBJ/EMBL/GenBank under accession numbers AB972403, AB972404, AB972405, AB972406, AB972407, and AB972408, respectively. The primer sequences for the constructed strains are described in the supplementary information (Supplementary Table 4).

### Media and culture conditions

*C. boidinii* cells were grown at 28°C on YPD medium (1% Bacto-yeast extract, 2% Bacto-peptone, 2% glucose) and SD medium (0.17% yeast nitrogen base without amino acids and ammonium sulfate, 2% glucose, and nitrogen sources). Nitrogen sources were (NH_4_)_2_SO_4_, KNO_3_, and CH_3_NHCl. The pH of SD medium was adjusted to 6.0 with NaOH. Growth was monitored by measuring the optical density at 610 nm (OD_610_).

### Construction of the *YNR1* and *AMO1* disruptant strains

The upstream region (1.1 kb) and downstream (1.3 kb) regions of the *YNR1* gene were amplified by PCR with the primer sets Fw-YNRu-*Bam*HI/Rv-YNRu-*Xho*I and Fw-YNRd-*Not*I/Rv-YNRd-*Bam*HI, respectively, using genomic DNA as a template. These PCR-amplified fragments were ligated into the *Xho*I-*Not*I backbone fragment of pBluescript II SK+/Zeocassette^TM^. The resultant vector (6.5 kb) was linearized with *Bam*HI and used to transform *C. boidinii* strain TK62 using a modified version of the lithium acetate method^47^. Zeocin-resistant colonies were selected on YPD medium supplemented with Zeocin^46^. The upstream (1.3 kb) and downstream (1.5 kb) regions of the *AMO1* gene were amplified by PCR with the primer sets Fw-AMOu-*Bam*HI/Rv-AMOd-XhoI and Fw-AMOd-*Not*I/Rv-AMOd- BamHI, respectively. These PCR-amplified fragments were ligated into the *Xho*I-*Not*I backbone fragment of pBluescript II SK+/Zeocassette^TM^. The resultant vector (6.9 kb) was linearized with *Bam*HI and used to transform *C. boidinii* strain TK62. The disruptions of these genes were confirmed by Southern blot analysis as previously described^48^.

### Generation of *C. boidinii* strains expressing Venus-Ynr1 fusion protein

A fragment of the 1.0-kb 5′ untranslated region and 2.7-kb *YNR1* coding region was amplified by PCR with the primer set Fw-YNR1-*Afl*III inf/Rv-YNR1-*Pst*I inf. The PCR product was fused with the 5.7-kb *Afl*III-*Pst*I fragment of pACT1-Venus^6^ to form pEX-Ynr1 by In-fusion HD ® cloning kit (TaKaRa, Kyoto, Japan). Next, a 9.4-kb fragment was amplified by inverse PCR with the generated plasmid as a template DNA, using the primer set Fw-YNR1-*Sac*I inv/Rv-YNR1-*Kpn*I inv. The Venus-coding region was PCR amplified with the primer set Fw-VENUS-*Kpn*I inf/Rv-VENUS-*Sac*I inf. These two fragments were fused to form pEX-Venus-Ynr1. The resulting plasmid was linearized with AfeI and then introduced into the wild-type host strain, *atg1Δ*, *atg11Δ*, and *atg30Δ* mutant strains.

### *VENUS-APE1* and *APE4-VENUS* expressions in *C. boidinii*

A backbone vector pACTL*-*Venus, encoding Venus under the control of the *ACT1* promoter with *LEU2*^48^ as Leucine auxotrophic marker was constructed by ligation of an AatII-*Nar*I fragment from pACT1-Venus^6^ with an *Aat*II-*Nar*I fragment of *LEU2* gene. For the construction of the plasmid for *VENUS-APE1* expression, a 1.3-kb *APE1* fragment was amplified by PCR with the primer set Fw-APE1-*Pst*I inf/Rv-APE1-*PstI* inf. Next, a 8.8-kb fragment was amplified by inverse PCR with the pACTL*-*Venus plasmid as a template DNA, using the primer set Fw-Tact-*Pst*I inv/Rv-VENUS-*Pst*I inv. These two fragments were fused to form pACTL-Venus-APE1 with the In-fusion HD ® cloning kit. The plasmid for *APE4-VENUS* expression, termed pACTL-APE4-Venus, was constructed from a *Sal*I-digested fragment of pACT1-Venus and an *APE4*-coding region that was amplified with the primer set Fw-APE4-*Sal*I inf/Rv-APE4-*Sal*I inf, using the In-fusion HD ® cloning kit.

### Construction of the *C. boidinii* *P_YNR-_* and *P_AMO_-*reporter strains

Putative promoter regions of *YNR1* and *AMO1* genes were amplified by PCR with the primer sets Fw-PYNR/Rv-PYNR and Fw-PAMO/Rv-PAMO, respectively. These 1.0-kb PCR-amplified fragments were fused with a fragment from pACT1-Venus digested with *Afl*III/*Sal*I, using the In-fusion HD ® cloning kit, which yielded two expression vectors of Venus under the regulations of *P_YNR_* or *P_AMO_* promoters.

### Host plant growth condition and yeast inoculation on plant leaves

*A. thaliana* plants were cultivated on mini-pots of cultivable lock-fiber (Nittobo) with Hoagland solution in growth chambers, as previously described^6^. *C. boidinii* cells were cultured to OD_610_ = 1.0. To follow yeast proliferation on the leaves of *A. thaliana*, 1 µl of yeast cell suspension (OD_610_ = 0.5) was spotted onto the upper sides of the leaves. To observe Venus and/or mCherry expression in *C. boidinii*, 5 µl of yeast suspension (OD_610_ = 0.5) was sprayed onto the upper sides of the leaves. To prepare the samples for qRT-PCR, yeast suspension was sprayed onto *A. thaliana* leaves and incubated for 5 days. For these experiments, we used leaves of two different ages. Young plant leaves, which were used in Figure 1, 2, 7, and S3, were growing leaves that had been grown for 2–3 weeks after the germination. Aged plant leaves, which were used in Figure 2 and 7, were wilting leaves that had been grown for 2–3 months after the germination.

### Comparison of yeast populations on *A. thaliana*

After *C. boidinii* cells were inoculated on the leaves of *A. thaliana,* 5 leaves were collected. Isolation of genomic DNA of *C. boidinii* and quantitation of the yeast populations on plant leaves were performed as previously described^6^.

### RNA preparation from cells grown on plants

Fifty to sixty leaves of *A. thaliana* onto which *C. boidinii* cells had been inoculated were harvested in tubes and frozen using liquid nitrogen. Total cellular RNA was extracted using ISOGEN, and qRT-PCR was performed as previously described^6^.

### Preparation of cell-free extract

*C. boidinii* cells were first cultured on SD supplemented with 7.6 mM ammonium for 12 h, shifted to SD containing with 7.6 mM of the indicated nitrogen source, and then collected 4 h after the medium transfer. Cells (approximately 100 OD_610_ units of cells grown in SD medium) were then resuspended in 0.1 M potassium phosphate buffer (pH 7.5) and disrupted with 0.5-mm zirconia beads in a Multi-Beads Shocker (YASUI KIKAI Co., Ltd, Osaka, Japan). Cell debris was removed by centrifugation at 15,000 rpm for 10 min at 4°C, and the resultant supernatant (cell-free extract) was used for enzyme assays. Protein concentrations were determined by the Bradford method^49^ using a protein assay kit (Bio-Rad Laboratories, Hercules, CA, USA), using bovine serum albumin as the standard.

### Enzyme assay

Ynr1 activity was measured according to Boer et al.^50^ with slight modifications. The assay mixture contained potassium phosphate buffer (final concentration 0.1 M, pH 7.5), 10 µL of 10 mM DTT, 10 µL of 1 mM FAD, 10 µL of 20 mM NADPH, and 250 µL cell-free extract in a final volume of 1 mL in a polystyrene cuvette (light path, 10 mm). The cuvettes were incubated for 2–3 min at 28°C, and the absorbance at 340 nm was monitored. The reaction was then initiated by the addition of 100 µL of 100 mM potassium nitrate, and the rate of decrease in absorbance at 340 nm was measured against a blank containing all the assay components expect potassium nitrate. Ynr1 activity is expressed in units (µmol/mg protein). Amo1 activity was measured as follows: the assay mixture contained potassium phosphate buffer (final concentration 0.1 M, pH 7.0), 15 µL of 50 mM 2,2′-azinobis-(3-ethylbenzthiazoline)- 6-sulfonate (ABTS), 15 µL of 100 units/mL horseradish peroxidase, and 100 µL of cell-free extract in a final volume of 1 mL in a polystyrene cuvette (light path, 10 mm). The cuvettes were incubated for 2–3 min at 28°C, and the absorbance at 405 nm was monitored. The reaction was then initiated by addition of 40 µL of 80 mM methylamine hydrochloride, and the rate of increase in absorbance at 405 nm was measured against a blank containing all assay components except methylamine hydrochloride. Amo1 activity is expressed in units (µmol/mg protein).

### Immunoblot analysis

To prepare samples for immunoblot analysis, cells were grown to A_610_ = 1.0 on nitrate medium, and then shifted to SD containing either 10 mM methylamine or no nitrogen source. Cells equivalent to 5 OD_610_ units were collected at the appropriate time points, washed once with water, suspended in lysate buffer containing 0.25 N NaOH and 150 mM β-mercaptoethanol, and incubated at 4°C for 10 min. Next, trichloroacetic acid (final concentration, 10%) was added. The samples were vortexed, incubated at 4°C for 10 min, and centrifuged. Subsequently, the pellet was washed twice with acetone and resuspended in a buffer containing 50 mM Tris-HCl, pH 7.5. The samples were denatured by boiling in SDS sample buffer, separated on 12% SDS-PAGE gels, and electrotransferred to nitrocellulose membranes. Blots were blocked for 1 h in 5% skim milk in TBS-T buffer (25 mM Tris, 220 mM NaCl, 27 mM KCl, 0.1% Tween 20, pH 8.0). These blots were incubated for 1 h with polyclonal anti-GFP antibody (Life Technologies) at 1:1000 dilution in TBS-T buffer, and then washed three times with TBS-T. Subsequently, blots were incubated for 1 h with anti–rabbit IgG antibody (life technologies) at 1:10000 dilution in TBS-T, and then washed again three times in TBS-T. Immunoreactive bands were detected with the Western Lightning Chemiluminescence Reagent Plus (Perkin-Elmer Life Science), and the signals were detected using the Light-Capture II system (ATTO). To ascertain the reproducibility, each of immunoblot analysis was conducted at least twice.

### Fluorescence microscopy

Confocal microscopy was carried out using a Zeiss LSM510 META/Axiovert 200 laser-scanning confocal inverted microscope equipped with a Plan Fluor 100×/1.45 NA oil objective. Venus fluorescence was obtained with a multiline (458, 477, 488, and 514 nm) argon laser and a 530–600 nm filter for emission. An HFT 405/514 beam splitter was used as a connecting filter. Fluorescence microscopy was performed using an IX81 inverted microscope (Olympus) equipped with an Uplan-Apochromat 100×/1.35 NA oil iris objective lens. Venus and mCherry signals were acquired using a Plan Fluor 100× lens (Carl Zeiss) with pinhole set to 1.02 airy units for YFP acquisition. Fluorescence observations of cells cultured *in vitro* were repeated at least twice and three shots at different fields were taken each time. For fluorescence microscopy of the cells on the plant surface, 3 different leaves were harvested for observation. At least five shots at different fields were taken per leaf.

## Supplementary Material

Supplementary Information Supplementary Information
