# Supplementary material for: Yeast nitrogen utilization in the phyllosphere during plant lifespan under regulation of autophagy
Source: Sci Rep. 2015 Apr 21;5:9719. doi: 10.1038/srep09719 (PMC5386246; doi:10.1038/srep09719)

Supplemental information

Title

Yeast nitrogen utilization in the phyllosphere during plant lifespan under regulation of autophagy

Authors and Affiliations

Kosuke Shiraishi[1], Masahide Oku[1], Kosuke Kawaguchi[1,*], Daichi Uchida[1], Hiroya Yurimoto[1] & Yasuyoshi Sakai[1,2,3]

[1] Division of Applied Life Sciences, Graduate School of Agriculture, Kyoto University, Kitashirakawa-Oiwake, Sakyo-ku, Kyoto 606-8502, Japan
[2] Research Unit for Physiological Chemistry, The Center for the Promotion of Interdisciplinary Education and Research, Kyoto University, Kyoto, Japan,
[3] Advanced Low Carbon Technology Research and Development Program, Japan Science and Technology Agency, K's Gobancho, 7 Gobancho, Chiyoda-ku, Tokyo 102-0076, Japan

Correspondence and requests for materials should be addressed to Y.S.
E-mail: ysakai@kais.kyoto-u.ac.jp
Phone: +81-75-753-6385
Fax: +81-75-753-6454

Supplementary Table 1

Ynr1 activity in the *ynr1Δ* strain and Amo1 activity in the *amo1Δ* strain.

Enzyme	Strain	Activity : units (μmol/mg protein)
Ynr1	WT	54.4
	ynr1Δ	n.d.*
Amo1	WT	178
	amo1Δ	n.d.

* n.d.: not detected

Average values of duplicate measurements are shown.

Supplementary Table 2

Ynr1 and Amo1 activities in response to various nitrogen sources.

Nitrogen source	Ynr1	Amo1
NH_4^+	2.0	n.d.*
NO_3^-	57	n.d.
$CH_3NH_3^+$	n.d.	152

* n.d .: not detected

Activity : units (μmol/mg protein)

Average values of duplicate measurements are shown.

Supplementary Table 3

Transcriptional level of *YNR1* and *AMO1* in response to various nitrogen sources.

Nitrogen source	*YNR1*	*AMO1*
NH_4^+	1.0*	1.0*
NO_3^-	30.7	4.9×10^2
$CH_3NH_3^+$	3.2	2.2×10^4

*Transcriptional level of *YNR1* and *AMO1* was first quantified using *ACT1* as the control, and then, the transcript level was expressed as the relative value to those in ammonium media.

Average values of duplicate measurements are shown.

Supplementary Table 4

List of oligonucleotide primers

Primer name	5'- sequence-3'
Fw-YNRu-*Bam*HI	GGATCCCCCAATTAGTACTAAACACGGGCG
Rv-YNRu-*Xho*I	CTCGAGGGAGTTATGTGCTGGTGAATAAAG
Fw-YNRd-*Not*I	GCGGCCGCCTCGCTTATAAAAATCTTTTTGTCTTGTC
Rv-YNRd-*Bam*HI	GGATCCCTAGAAGGATATTGTATATGAGGTG
Fw-AMOu-*Bam*HI	GGATCCCTATTGAAAGGGAATTGCCTATCC
Rv-AMOd-*Xho*I	CTCGAGCCTAATTAGGTATATCAATCCACC
Fw-AMOd-*Not*I	GCGGCCGCGTCTTATATTTTTTCTTCTTCATC
Rv-AMOd-*Bam*HI	GGATCCCTAGCTCTGATGTCTTAAAGTTATC
Fw-ATG11u-*Pst*I	CTGCAGGCATTGCTTCTTTGGGAC
Rv-ATG11u-*Not*I	GCGGCCGCTTTGTCTATGAGTTGAC
Fw-ATG11d-*Xho*I	CTCGAGGACAATGCAGTTGACCG
Rv-ATG11d-*Pst*I	CTGCAGCAACGAAGATGCCAACTTG
Fw-ATG17u-*Eco*RI	GGAATTCCTCTGTTAAACAATAAATCCGTAGCC
Rv-ATG17u-*Bam*HI	CGGGATCCGTAATTCTCAAATTCATGG
Fw-ATG17d-*Cla*I	CCATCGATCTATGTACATGATATATATATAATATAT
Rv-ATG17d-*Eco*RI	GGAATTCCAATTTAAAAGATTGTACTGTGATAG
Fw-YNR1-*AflIII* inf	CTTTTGCTCACATGTGACTTCATATTGGAAAATGCCACTAAAATT
Rv-YNR1-*PstI* inf	GATTAAATTCCCTGCAGTTAAAAAGCAACACAGTATTCTTCACCAAA
Fw-YNR1-*SacI* inv	GAGCTCATGATTGTAGCATCAGGTACAGAT
Rv-YNR1-*KpnI* inv	GGTACCTATGAATTATATTTGGAGTTATGTGCTGGT
Fw-VENUS-*KpnI* inf	TAATTCATAGGTACCATGGTTTCTAAAGGTGAAGAATTATTCA
Rv-VENUS-*SacI* inf	TACAATCATGAGCTCTTTATATAATTCATCCATACCTAAAGTAATACC
Fw-APE1-*Pst*I inf	AATTATATAAACTGCAGATGGTTGGTGCTCACACAGATT
Rv-APE1-PstI inf	GATTAAATTCCCTGCAGCTAATCTTGGATACCATCGACTAAAATTTTTGGT
Fw-Tact-*Pst*I inv	GAGCTCCTGCAGGGAATTTAATC
Rv-VENUS-*Pst*I inv	TTTTCTGCAGTTTATATAATTCATCCATACCTAAAGTAATACCAGC
Fw-APE4-*Sal*I inf	ATATTACAAAAGTCGACATGGTTGGTGCTCACACAGATT
Rv-APE4-*Sal*I inf	TTAGAAACCATGTCGACAAATTTTGGTTCTAATTCATTGTATCTTTCGAAATATTG
Fw-PYNR	TGGCCTTTTGCTGGCCTTTTGCTCACATGTCTTGCAGTCTAAAAATAAACA
Rv-PYNR	CACCTTTAGAAACCATGTCGACTTATGAATTATATTTGGAGTTAT
Fw-PAMO	CTTTTGCTCACATGTAGTAAAGACGGACAACTATAATTGA
Rv-PAMO	CTTCACCTTTAGAAACCATGTCGACTTTTATGATATTATATAATAAAG

Legends to Supplementary Figures.

Supplementary Figure 1 | Comparison of amino-acid sequences of nitrate reductase and amine oxidase. (**a**) Alignment of CbYnr1 and HpYnr1. *YNR1* contains a 2667-bp ORF encoding a protein of 889 amino acids (Ynr1). The predicted amino acid sequence of *YNR1* has a high degree of identity (56%) to nitrate reductase of the yeast *H. polymorpha*. (**b**) Alignment of CbAmo1 and HpAmo1. *AMO1* contains a 2112-bp ORF encoding a protein of 704 amino acids (Amo1) with significant identity (76%) to amine oxidase of *H. polymorpha*. Close to the N terminus, Amo1 has a peroxisomal targeting signal type 2 (PTS2) motif.

Supplementary Figure 2 | Molecular cloning and disruption of the *YNR1* and *AMO1* genes. Growth of the *C. boidinii* wild-type, *ynr1Δ*, and *amo1Δ* strain on SD medium containing (**a**) 3.8 mM $(NH_4)_2SO_4$, (**b**) 7.6 mM CH_3NH_3Cl, and (**c**) 7.6 mM KNO_3. Symbols: ○; wild-type, ■; *amo1Δ*, ▲; *ynr1Δ*.

Supplementary Figure 3 | mRNA levels of *YNR1* during the daily light–dark cycle. Transcript level of *YNR1* is expressed as the relative value to the sample collected at 4 h. Gray bars indicate the dark period. Average values of duplicate experiments are shown.

Supplementary Figure 4 | Standard curve for Venus fluorescence intensity vs. methylamine concentrations. A *C. boidinii* strain expressing Venus under the control of *AMO1* promoter (the PAMO strain) was used for measurement of local methylamine concentrations. Cells were inoculated onto SD-agar plates containing different concentrations of methylamine (10^{-4} – 1 mM). At four hours after the inoculation, *C. boidinii* cells were collected and observed by fluorescence microscopy. The cellular fluorescence intensities were proportional to the methylamine concentration in agar plates within the range 10^{-3} – 10^{1} mM. The fluorescence intensities were measured from at least 50 cells and averaged. Error bars show standard deviations of 5 shots of different fields. The Similar results were obtained from three independent experiments.

Supplementary Figure 5 | Ynr1 transport to the vacuole is selective. (**a**) Immunoblot analysis of Venus-tagged Ynr1 in *atg8Δ* cells transferred from nitrate to methylamine medium. (**b**) Immunoblot analysis of Venus-tagged Ynr1 in *atg17Δ* cells transferred from nitrate to methylamine medium. (**c**) Fluorescence images of Venus-Ynr1–expressing *C. boidinii atg8Δ* and *atg17Δ* cells during the nitrogen-source shift from nitrate to methylamine. Bar, 2μm. (**d**) Quantitation of Venus-Ynr1 puncta per cell, estimated from the fluorescence image analysis in (**c**). For each sample, at least 50 cells were analyzed. Error bars show standard deviations of the counted dot number per cell. (**e**) Fluorescence image of cells expressing Venus under the control of the *ACT1* promoter.

Supplementary Figure 6 | Co-localization of mCherry-Ynr1 and other Cvt vesicle proteins. (**a**) Fluorescence images of cells expressing both mCherry-Ynr1 and Venus-Atg8. Yellow arrows indicate co-localization of Atg8-Venus and mCherry-Ynr1. Bar, 2 μm. (**b**) Fluorescence images of both mCherry-Ynr1– and Ape4-Venus–expressing cells. Yellow arrows indicate co-localization of Ape4-Venus and mCherry-Ynr1. Bar, 2 μm.

Supplementary Figure 1a

```
CbYnr1      MIVASGTDHLQLDGTASDNSFVLETKIKDERSELEELSKRFNIPVLLDGPPRTEVLELDKKTKDYHVARNPELIRLTGTHPFNCEAPLTT
HpYnr1      -----------MDSVVTEVTYGLEIKKIKEITELP-------FPVRQD-SPLSEVLPTDLKTKDNFVARDPDLLRLTGSHPFNSEPPLAK
                       :*...:: :: ** *  .* :**        :**  * .* :***  * **** .***:*:*:****:****.*.**:.

CbYnr1      LYNSGFLTPAELHYVRNHGPAPKVEDSEILDWEITIDGMVEKPYKLTLREIMETLDIFTTPVTFCCAGNRRKEQNMVKKGKGFNWGAAGI
HpYnr1      LYDSGFLTPVSLHFVRNHGPVPYVPDENILDWEVSIEGMVETPYKIKLSDIMDQFDIYTTPVTMVCAGNRRKEQNMVKKGTGFNWGAAGT
            **:******..**:******.* * *.:*****::*:****.***:.* :**: :**:*****: ***************.********

CbYnr1      STSLWTGPMLADIIAKAIPSKKARFVWMEGGDDPAKGPYGTCVRLAWIMDPERSIMLAYKQNGQLLTPDHGRPLRVVIPGVIGGRSVKWL
HpYnr1      STSLWTGCMLGDVIGKARPSKRARYIWMEGADNPANGAYGTCVRLSWAMDPERCIMMAYKQNGEWLHPDHGKPLRVVIPGVIGGRSVKWL
            ******* **.*:*.** ***:**::****.*:**:*.*******:* *****.**:******: * ****:******************

CbYnr1      KKSIVMDRPSENWYHYFDNRVLPTMVTPEMASADESWWKDERYALYDLNIQSVTCKPECGETLVVDDKEKDFTIKGFAYNGGGVRVGRVE
HpYnr1      RKLVVSDRPSENWYHYFDNRVLPTMVTPEMAKSDDRWWKDERYAIYDLNLQTIICKPENQQVIKISDDE--YEIAGFGYNGGGIRIGRIE
            :* :* *************************.:*: ********:****:*:: ****  :.: :.*.*  : * **.*****:*:**:*

CbYnr1      VSLDKGVTWRLAEIDYPEDRYREAGYFRMFGGLVNICDRLSCLCWCFWEIKVKTGELLNAKDIVVRAMDERMCVQPRNMYWNVTSMLNNW
HpYnr1      ISLDKGKTWKLTEIDYPEDRYREAGYFRLFGGLVNVCDRMSCLCWCFWKLKVPLSELATSKDILVRGMDERMVVQPRTMYWNVTSMLNNW
            :***** **:*:****************:******:***:********::**  .** .:***:**.***** ****.***********

CbYnr1      WYRVAIVKLEENVIKFEHPCRPNTDGGWMDRVKDEGGDILDNNWGEEVGDAEDSHKRVKKPKVDEDLLLMCNPEKVNNIITIKEFESHKD
HpYnr1      WYRVAIIR-EGDALRFEHPVVANKPGGWMDRVKAEGGDILDNNWGEVDDTVKQAER---KPRVDEDIEMMCNPEKMDVIIKYSEFEAHKD
            ******:: * :.::****  .*. ******** ************  . .:::.:   **:****: :******:: **. .***:***

CbYnr1      DAVNPWFEVKGHIFNGAEYLDDHPGGRESIINMAGEDATDDFLAIHSDSAKKLIQQWHLGKLETSGASDNAATANVVKELTPTLLDTKKW
HpYnr1      SETEPWFAVKGHVFDGSSYLEDHPGGAQSILMVSGEDATDDFIAIHSSYAKKLLPPMHLGRLEEVSSVTKVKSVEENVKR-EVLLDPRKW
            . .:*** ****:*:*:.**:***** :**: ::********:****. ****:   ***:**  .:  :. :.:   :   .***.:**

CbYnr1      KAIQLTEREQISPDTIIFHFALEHKDQQVGLNVGNHIYLRLKDKEGKFVMRAYTPVTSNRMKGTLGVLIKLYLPKGDFP-GGKLTTLLND
HpYnr1      HKITLAEKEIISSDSRIFKFDLEHPEQLIGLPTGKHLFLRLKDSSGKYVMRAYTPKSSNSLRGRLEILIKVYFPNREYPNGGIMTNLIEN
            : * *:*:* **.*: **:* *** :* :** .*:*::*****..**:******* :** ::* * :***:*:*: ::* ** :*.*:::

CbYnr1      LAIGSYAETKGPIGEFQYKGFGNCIYKKKEYKVKHFLQVAGGSGITPPFQIIQEVHYLITSGESKEEPTMDLFFGNRTEADILCKAQLDA
HpYnr1      LQVGNQIEVKGPVGEFEYVKCGHCSFNNKPYQMKHFVMISGGSGITPTYQVLQAIFS-----DPEDTTSVQLFFGNKKVDDILLREELDC
            * :*.  *.***:***:*   *:* .::* *::***: ::*******.:*::* :.      :.:: .:::*****:.  *** : :**.

CbYnr1      MQKDIGEDKFRINYNISTLPEVCAPNY-TTGRLSANDLAKYVEGYKPGEMMILLCGPPPMVKMVIDWAIQTGFGEEYCVAF
HpYnr1      LQIKHP-EQFKVDYSLSDLHHLPENWSGLKGRLTFNILDSYVQGKNMGEYMLLVCGPPGMNGVVENWCKARNLDKQYVVYF
            :* .   ::*:::*.:* * .:       .***: * * .**:* : ** *:*:**** *  :* :*.    .:.::* * *
```

Supplementary Figure 1b.

```
CbAmo1      MERLAQISSQTTGSIAAPSRPAHPLDPLSIEEISAITAVVKNHFAGRQISFNTVTLREPTKKAFLEWKEQGGAFPPRNAYYVILEAGVPG
HpAmo1      MERLRQIASQATAASAAPPRPAHPLDPLSTAEIKAVTSTVKSYFAGKQISFNTVTLREPARKAYIQWKEQGGPLPPRLAYYVILEAGKPG
            **** **:**:*.: ***.**********  **.*:*:.**.:***:************::**:::******.:*** ********* **

CbAmo1      VKEGIVSVNNLSVIEVKSLEQVQPILTVEDLISTEDIIRKDPRVIEQCVISGIPANEMHKVYCDPWTIGFDERWGAGRRLQQALMYYRSD
HpAmo1      VKEGLVDLASLSVIETRALETVQPILTVEDLCATEDVIRNDPAVIEQCVLSGIPANEMHKVYCDPWTIGYDERWGTGKRLQQALVYYRSD
            ****:*.: .*****.::** ********** :***:**:** ******:*******************:*****:*:******:*****

CbAmo1      EDDSQYSHPLDFCPIVDTEEKKVIFIDVPNRRRKVSKHKHSNFNPKDMIEKYGTLRTDGKPIDILQPEGVSFKMDGNVISWSNFNIHIGF
HpAmo1      EDDSQYSHPLDFCPIVDTEEKKVIFIDIPNRRRKVSKHKHANFYPKHMIEKVGAMRPEAPPINVTQPEGVSFKMTGNVMEWSNFKFHIGF
            ***************************:************:** **.**** *::*.:. **:: ********* ***:.****::****

CbAmo1      NYREGIVLSDISYNDHGNVRPLFHRISLSEMIVPYGSPDFPHQRKHALDIGEYGAGYMTNPLALGCDCKGVIHYLDAHFADRAGDPITVK
HpAmo1      NYREGIVLSDVSYNDHGNVRPIFHRISLSEMIVPYGSPEFPHQRKHALDIGEYGAGYMTNPLSLGCDCKGVIHYLDAHFSDRAGDPITVK
            **********:**********:****************:***********************:****************:**********

CbAmo1      HAVCIHEEDDGLLQKHSDFRDNFATSIVTRATKLIISQIFTAANYEYCIYWVFMQDGTIKLDVKLTGILNTYVLADGEESGPWGTKVYPG
HpAmo1      NAVCIHEEDDGLLFKHSDFRDNFATSIVTRATKLVISQIFTAANYEYCLYWVFMQDGAIRLDIRLTGILNTYILGDDEEAGPWGTRVYPN
            :************ ********************:*************:********:*:**::********:*.*.**:*****:***.

CbAmo1      VNAHNHQHLFALRLHPRIDGDGNSVCTSDACSAEAPVGSPENMYGNGFYAKRTVFKTVADSETNYESSTGRTWDFFNPKKLNPYSGKPVS
HpAmo1      VNAHNHQHLFSLRIDPRIDGDGNSAAACDAKPSPYPLGSPENMYGNAFYSEKTTFKTVKDSLTNYESATGRSWDIFNPNKVNPYSGKPPS
            **********:**:.*********..:.** .:  *:*********.**:::*.**** ** *****:***:**:***:*:******* *

CbAmo1      YKLVSAQCPPLLAKPGALVYKRAPWANTTIKVVPFKEDRLYPSGDHVPQWSGDGNIGMRKWLGDKTDKIEDTDILVFHTFGISHFPAPED
HpAmo1      YKLVSTQCPPLLAKEGSLVAKRAPWASHSVNVVPYKDNRLYPSGDHVPQWSGDGVRGMREWIGDGSEKIENTDILFFHTFGITHFPAPED
            *****:******** *:** ******. :::***:*::****************  ***:*:** ::***:****.******:*******

CbAmo1      FPLMPAEPISLLMRPRHFFTENAGMDIVPSHAMTTTEARKATNLEVTSDTDKSSKLAFETTSACCGSTRFVKEK
HpAmo1      FPLMPAEPITLMLRPRHFFTENPGLDIQPSYAMTTSEAKRAVHKEAK---DKTSRLAFEGS--CCGK-------
            *********:*::*********.*:** **:****:**::*.: *..    **:*:**** :  ***.
```

Supplementary Figure 2.

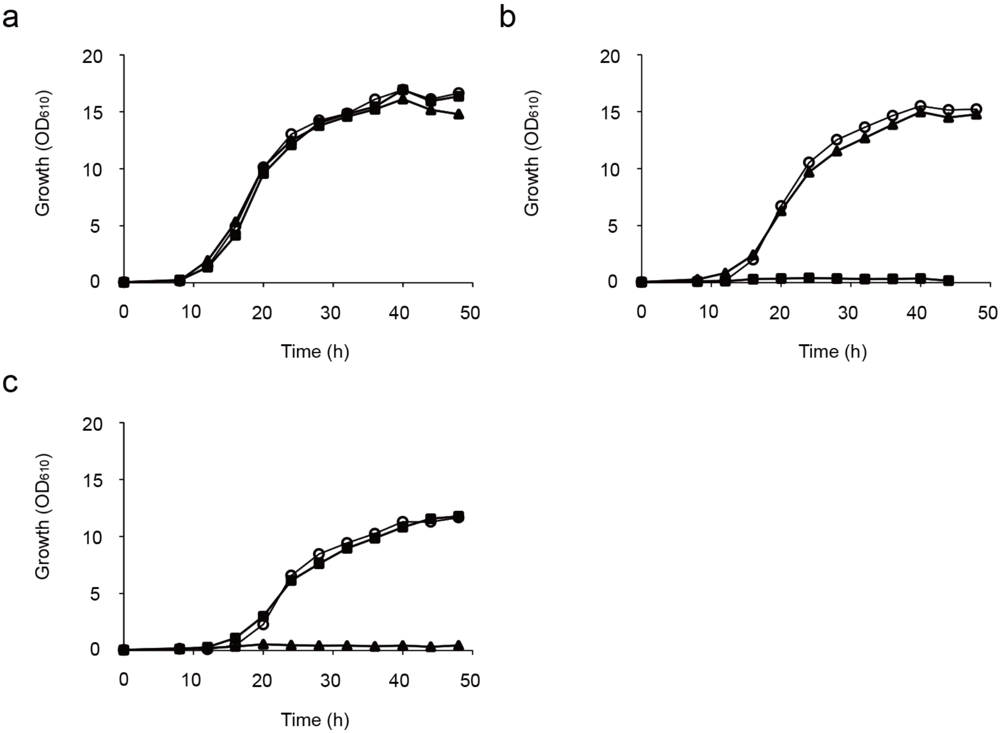

Supplementary Figure 3.

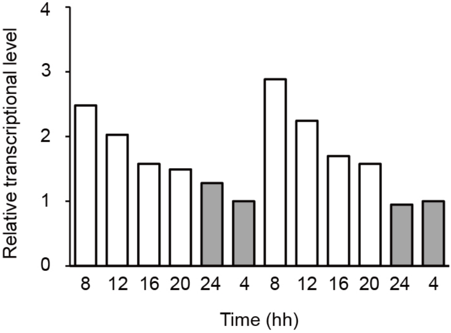

Supplementary Figure 4.

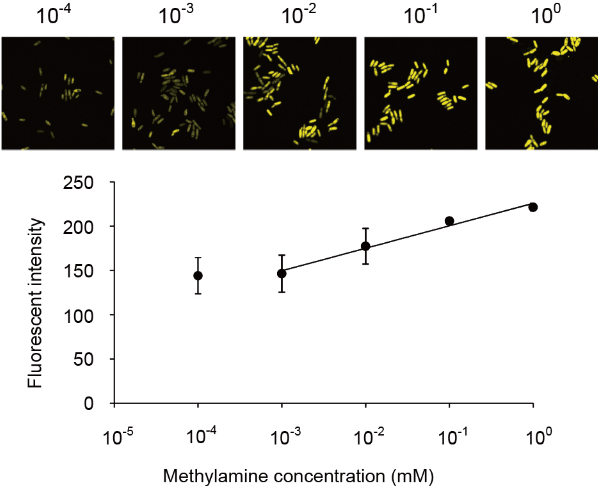

Supplementary Figure 5.

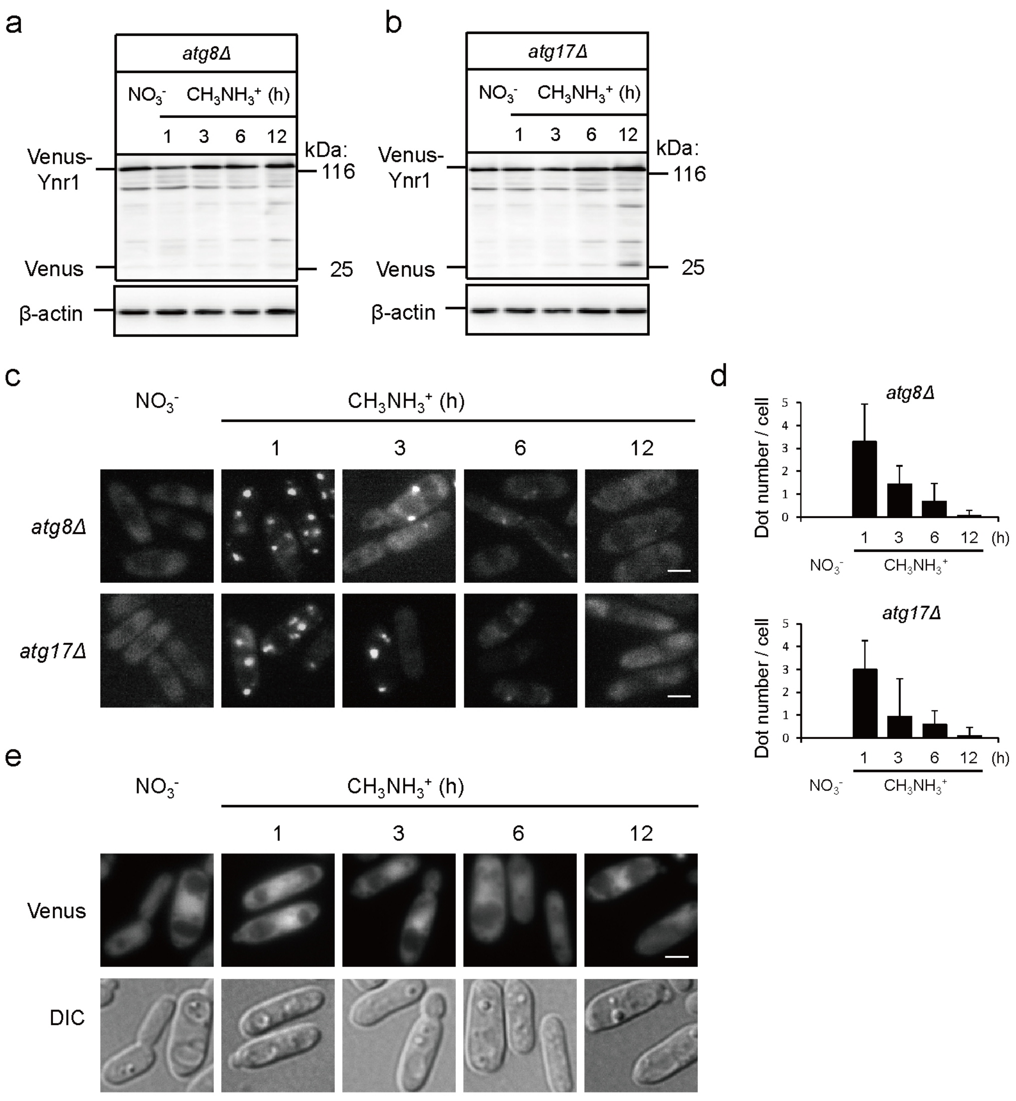

Supplementary Figure 6.

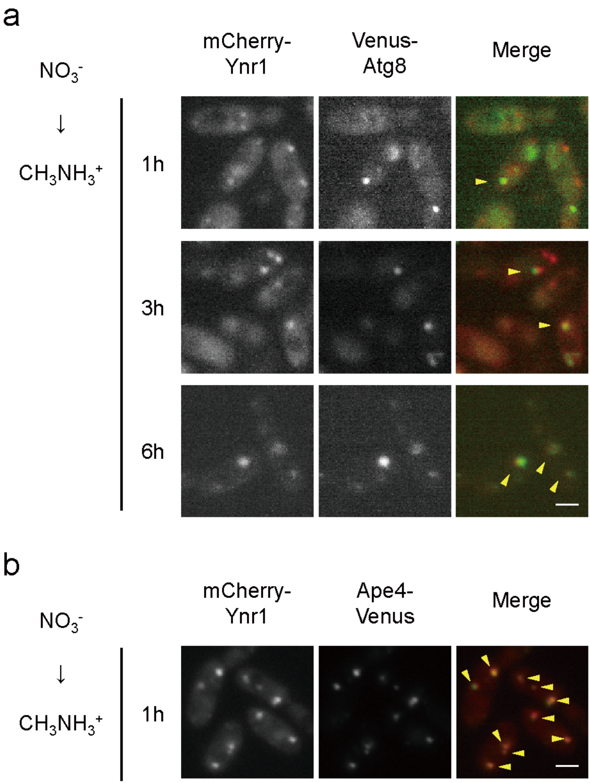

Supplement: Supplementary Information [file srep09719-s1.pdf]
